# Efficacy of transcutaneous electrical nerve stimulation (TENS) in the management of trigeminal neuralgia: A systematic review and meta-analysis

**DOI:** 10.4317/jced.60500

**Published:** 2023-06-01

**Authors:** Mukta Motwani, Aditi Fadnavis, Apeksha Dhole

**Affiliations:** 1 Department of Oral Medicine and Radiology, Vidya Shikshan Prasarak Mandal Dental College & Research Center (VSPM DCRC), Nagpur, India; 2 PG student Department of Oral medicine and Radiology , VSPM dental college and Research Centre, Nagpur, India; 3 V.S.P.M. Dental College and Research Center, Nagpur, India

## Abstract

**Background:**

Trigeminal Neuralgia is one of the most painful disorders known to man. So making patient pain free and to achieve a better quality of life in TN patients is one of the biggest challenge. Non-invasive procedures like Transcutaneous electrical nerve stimulation (TENS) have been tried clinically for Trigeminal neuralgia. Aim: The systematic review and meta-analysis aimed to compare and evaluate the efficacy of transcutaneous electric nerve stimulation in the management of trigeminal neuralgia. The present review has been registered with PROSPERO – An international prospective register of systematic review CRD42021254136.

**Material and Methods:**

An electronic search was done in PubMed, Cochrane Library, Science Direct, Google Scholar, EBSCOHOST. The assessments of articles were done using selection criteria and PRISMA guidelines Only prospective clinical trials like Randomized clinical trials (RCTs) and clinical trials were included in this review. A total of three studies were included in the meta -analysis,

**Results:**

The proportion of total number of patients after TENS therapy across studies with p-value < 0.0001 for each which showed statistically significance. The overall difference in two groups was significant with standardize mean difference of 3.03 [95% CI: 2.50, 3.56].

**Conclusions:**

TENS can be an effective treatment modality for trigeminal neuralgia in reducing the pain intensity with no reported side effects for patients with trigeminal neuralgia alone or even in combination with other first line drugs.

** Key words:**TENS, TN, TENS, TN, Transcutaneous electrical nerve stimulation.

## Introduction

Neuralgia (Greek neuron, “nerve” + algos, “pain”) is pain in the distribution of a nerve or nerves. Among all the types of neuralgias, Trigeminal neuralgia is one of the most common neuralgia and painful disorder of the orofacial region (1).

TN being a debilitating disease, making patient pain free and good quality of life in patients is one of the biggest challenge. Many treatment modalities have been described for TN from time to time. Conventional modalities have major disadvantage leading to reduced patient compliance (2,3). Hence, attempts have been made to use non- invasive procedures like TENS which can be used alone or as an adjuvant.

TENS uses electric current to activate nerves in order to decrease pain. Studies have reported that TENS is a promising and safe modality for TN with increased patient compliance(4). This systematic review and meta- analysis discusses about the efficacy of TENS in trigeminal neuralgia.

-Structured question 

The question that needs to be assessed in this review is –

Is transcutaneous electrical nerve stimulation (TENS) effective in treatment of trigeminal neuralgia? 

## Material and Methods

-Literature search 

The present review was carried out following the Preferred Reporting Items for Systematic Reviews and Meta-Analyses (PRISMA) criteria (http://www.prisma statement.org) and was registered on PROSPERO on 11/06/2021 (International prospective register of systematic reviews) Protocol number of the present systematic review is CRD42021254136.

 An electronic search was done in PubMed, Google Scholar, Google Scopus, EBSCOhost, Web of science, Cochrane library. Articles published from year 2010 to year 2020 articles in English language. Searches were prior to final analysis.

**Figure 1:**
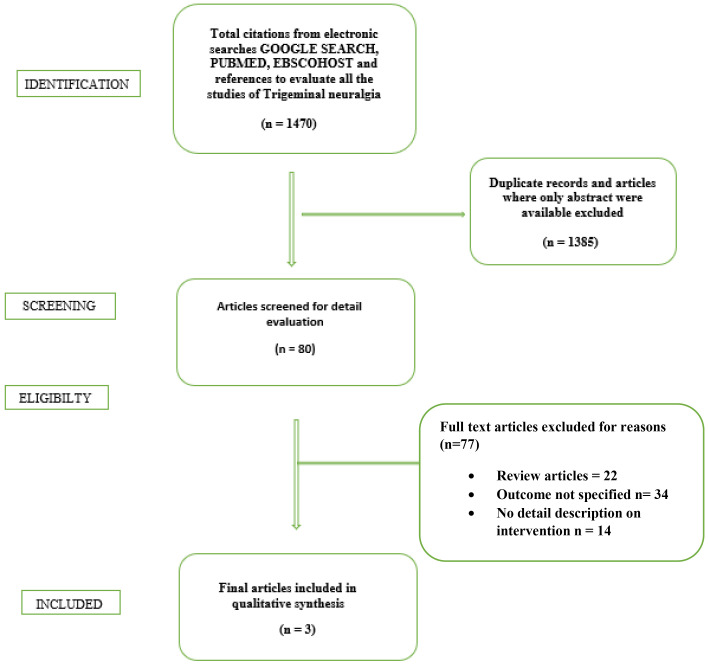
Flow chart showing systematic literature search for the present review.

The search strategy included for PubMed was – “TENS and Trigeminal Neuralgia”, “Neuralgia and TENS”, “Transcutaneous electrical nerve stimulation”. References in full text articles were manually searched additionally (Fig. 1)

-Study selection criteria 

Studies that did not comply with the inclusion criteria that did not have full text, different languages, animal studies were all removed. Two independent authors screened the initial titles and abstracts to find all the eligible studies. The full texts were retrieved according to the inclusion and exclusion criteria. All differences of opinions were discussed and resolved in consultation with another independent author.

-Data collection and analysis

The data was collected from the studies that were included based on the author’s name, publication year, study design, subjects, intervention, treatment duration, method of pain intensity measured, and outcome assessed.

-Selection of studies

The data of studies that assessed transcutaneous electric nerve stimulation in patients with trigeminal neuralgia were considered. If studies had similar parameters, the relevant data was described and synthesized.

-Data extraction and management

A Preferred Reporting Items for Systematic Reviews and Meta-Analyses study flow diagram was used to document the screening process.

Inclusion Criteria was *in vivo* studies, study designs like Randomized controlled trials (RCTs) , prospective clinical trials where Transcutaneous electrical nerve stimulation was used as a treatment modality for trigeminal neuralgia , Original articles, articles in English language.

Exclusion Criteria excluded studies with unavailable or incomplete data, clinical case reports, case series, literature review, abstracts, other language articles, books, reports, animal studies, letter to the editor, studies that are non-randomised, studies of experimental pain, clinical observations or systematic reviews.

The present systematic review, 1470 articles were searched on electronic database; duplicate articles were removed and eligibility were checked of 80 articles. 77 articles were excluded as they not meet the inclusion criteria and did not specifically described detailed description of interventions. Finally, meta -analysis was carried out after selecting 3 articles.

-Risk of Bias Assessment

The evaluation of methodological quality was carried out in duplicate and independently by two reviewers (AF and MM) according to the guidance in Higgins, JPT, Green, S (editors): the Cochrane Handbook for systematic review of Interventions Versions 5.1,0 (updated March 2011) The Cochrane collaboration, 2011.

-Statistical Analysis

The statistical analysis of present systematic review was carried out by R software version 3.5.3. Cochran’s Q test is the test used for heterogeneity in meta- analyses. The variation in the study outcome is determined by heterogeneity i.e. I2. The formula applied to calculate the percentage of variation across the study described by I2 is: I² = 100% × (Q-df)/Q.

## Results

The present systematic review and meta analysis aimed to evaluate the efficacy oftranscutaneous electrical nerve stimulation in treatment of trigeminal neuralgia. A total of 3 studies were included in the metaanalysis wich were obtained from different data bases through a systematic search.

Before TENS therapy, there were 71 patients. The estimates of measures of heterogeneity for number of patients before TENS therapy based on three studies were: I2 = 0% with confidence Interval (0% - 89.6%) and H=1 (95% CI: 1.00-3.10). The presence of homogeneity resulted into a Q-statistic of 0.15 indicating statistical insignificance with *p-value* of 0.9299 for 2 degrees of freedom. the total number of patients before TENS therapy and number of patients after TENS therapy across three studies is shown in Table 1.

**Table 1:**
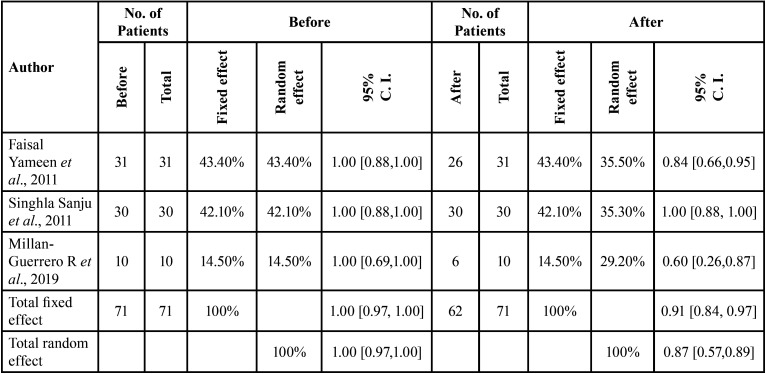
Comparison of number patients before TENS therapy and number of patients improved after TENS therapy across different studies.

The estimates of measures of heterogeneity for number of patients after TENS therapy based on three studies were: I2 = 85.4% with confidence Interval (57.3% - 95%) and H=2.62 (95% CI: 1.53 - 4.49). The presence of heterogeneity resulted into a Q- statistic of 13.74 indicating statistical significance with *p-value* of 0.0010 for 2 degrees of freedom. The proportion of total number of patients after TENS therapy across studies calculated fixed effects and random effect were 0.9191 (95% CI: 0.8361-0.9782) and 0.8771 (95% CI: 0.5861-1.0000) with *p-value* < 0.0001 for each which showed statistically significance 

TENS was effective in increasing number of pain-free patients across all the three studies due to significant fixed and random effects.

**Figure 2:**
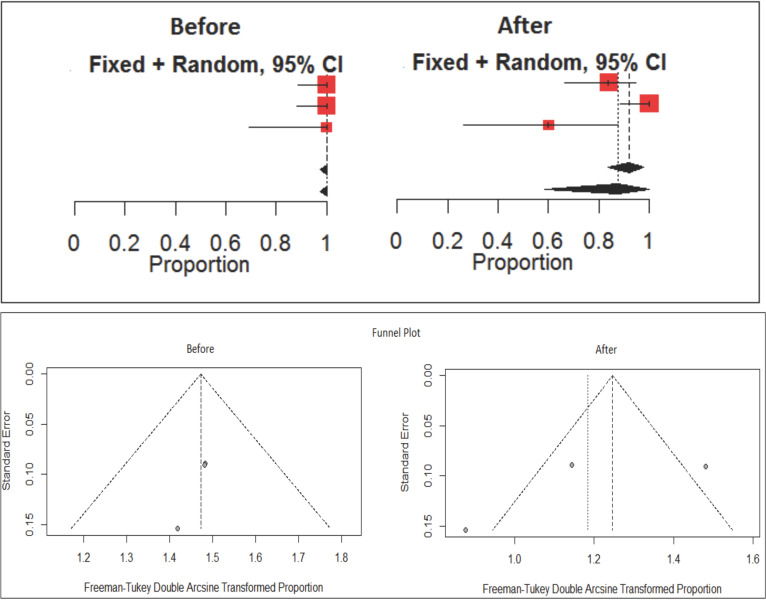
Forest plot and funnel plot showing the proportion of number of patients according to TENS therapy in different studies.

The forest plot provides the visualization of proportion across studies. Each study is represented by a line in the plot. The event rates of each study are listed. Effect estimates and weights of each study given in the Table. All this information presented in the forest plot. If the weight is bigger of the study the box is also bigger, smaller the box then smaller the weight. Each line represents the 95% confidence interval for that particular study (Fig. 2).

Study by Millan-Guerrero R *et al*., 2019 contributed heterogeneity to the parameter, while the other 2 studies showed lower heterogeneity with I2 value of 0% and corresponding *P*-value of 0.3359 shown in Table 2 represented by VAS score.

**Table 2:**
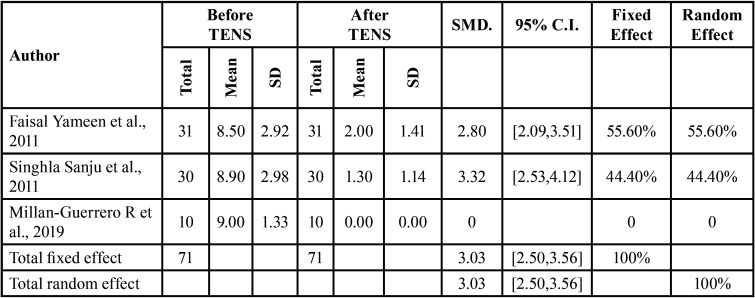
Comparison of VAS score before and after TENS therapy across different studies.

**Figure 3:**
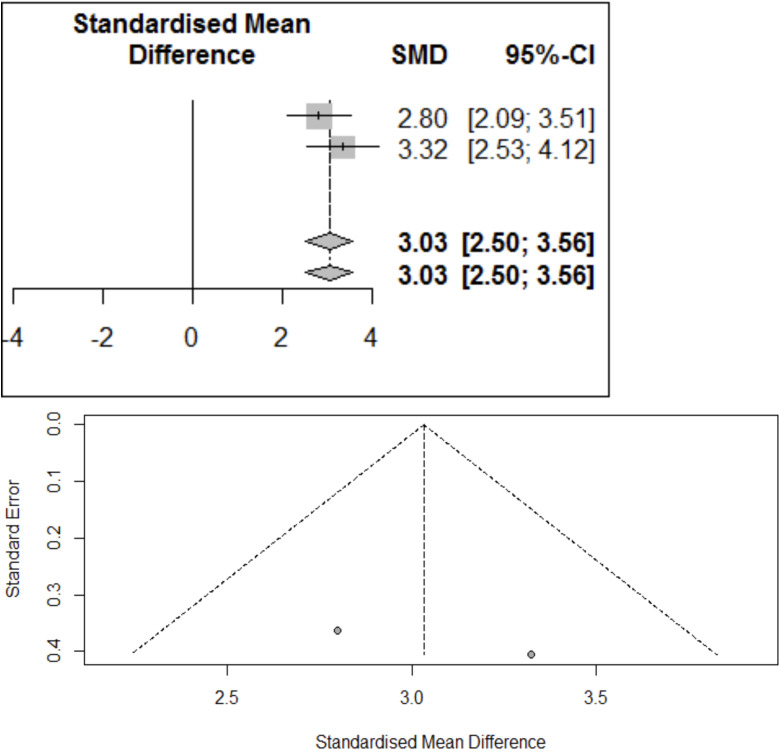
Forest plot and funnel plot showing the effect of VAS score in different studies.

The overall difference in two groups was significant with standardize mean difference of 3.03 (95% CI: 2.50, 3.56) which is significant with the corresponding *P*-value < 0.0001. The parameter showed significant homogeneity across studies with I2 of 0% and *P*-value of 0.3359. A forest plot of mean difference of VAS score is shown in Figure 3.

This signifies that TENS was effective in reducing the pain intensity when measured by VAS score as the overall difference in two groups i.e. before and after TENS therapy was significant.

A funnel plot was graphed to assess the possible publication bias in the study (Fig. 3). Egger’s test is a linear regression of the intervention effect estimates on their standard errors weighted by their inverse variance. Test result t = 0.173 with indicating possible publication bias is present.

## Discussion

TENS is a non- invasive, safe, simple, effective method for reducing pain. TENS relieves pain through selective activation of non-noxious, low threshold, large diameter A beta fibres which in turn inhibits ongoing central nociceptive cell activity and reduce central sensitization in the spinal cord (5).

In the study by Yameen *et al*. compared two different modes of TENS i.e. constant mode and burst mode for three weeks duration. They found significant improvement in 26 patients out of 31 and the VAS score was reduced significantly from mean score of 9 to 2.0 in group with constant mode of TENS (6). TENS was effective in both the modes while constant being more effective than the burst mode, while use of only burst mode by Singhla *et al*. in 30 patients of TN showed significant improvement in reduction of pain intensity (7).

The study conducted by Guerrero *et al*. TENS was compared with carbamazepine for its effectiveness in refractory TN patients in which 60 % patients improved significantly after 1 month interval and the VAS score also significantly reduced from mean score of 9.0 ± 1.333 to 0.3 ± 0.949 as compared to carbamazepine group which showed 50% patients showed significant improvement and the VAS score reduced from 9.40 ± 1.075 0.470 to 2.2 ± 1.61 after 2 months. This shows that TENS is an effective modality in patients with refractory TN (8).

Of all the included publications, the study by Guerrero *et al*. contributed heterogeneity to the parameter hence it was not included in the funnel plot. The funnel plot was graphed with remaining two studies which indicated possible publication bias present with a t value of 1.0173.

Pain was the primary concern for all the patients in these three included studies. TENS was reported to relieve pain significantly across studies. The proportion of number of pain free patients after TENS therapy showed statistically significant difference (*p* < 0.001). Taking into consideration the reduction in pain intensity across the three studies the standardized mean difference was statistically significant.

The results put forwarded by this meta-analysis suggests that TENS can be an effective treatment modality with no reported side effects for patients with trigeminal neuralgia alone or even in combination with other first line drugs.

## Conclusions

TENS was found to be effective in significantly reducing VAS score and increasing number of pain free patients. Therefore, it could be a treatment modality for trigeminal neuralgia as it is easy to use, non- invasive, cost effective and safe with no side effects. Also it can be used in patients who cannot tolerate medications and who have failed to respond adequately to other treatment regimens or have unbearable side effects.

## Limitations

Very few studies are available in the literature where TENS was used as a treatment modality in managing trigeminal neuralgia so only 3 studies are included in the meta -analysis. Patient population were not the same across studies with randomised controlled trials and comparison of TENS with other modalities was lacking in two of the three studies.
